# Development and evaluation of a 3D ensemble framework for automatic diagnosis of early osteonecrosis of the femoral head based on MRI: a multicenter diagnostic study

**DOI:** 10.3389/fsurg.2025.1555749

**Published:** 2025-02-14

**Authors:** Miao Yang, Fuchou Hsiang, Chengfan Li, XiaoYi Chen, Changqing Zhang, Guangchen Sun, Qiliang Lou, Wenhui Zhu, Hongtao Zhao, Feng Liu, Xuehai Ding, Jun Xu

**Affiliations:** ^1^ School of Computer Engineering and Science, Shanghai University, Shanghai, China; ^2^ Department of Orthopedic Surgery, Shanghai Sixth People’s Hospital Affiliated to Shanghai Jiao Tong University School of Medicine, Shanghai, China; ^3^ Ningbo Institute of Life and Health Industry, University of Chinese Academy of Sciences, Ningbo, China; ^4^ Department of Orthopaedics, The First People’s Hospital of Jiashan, Zhejiang, China; ^5^ Department of Orthopaedics, Sanmenxia Central Hospital, Sanmenxia, China; ^6^ Department of Orthopaedics and Traumatology, Xinghua Traditional Chinese Medicine Hospital, Xinghua, China

**Keywords:** MRI, osteonecrosis of the femoral head, artificial intelligence, predictive model, clinical decision-making

## Abstract

**Background:**

Efficient and reliable diagnosis of early osteonecrosis of the femoral head (ONFH) based on MRI is crucial for the formulation of clinical treatment plans. This study aimed to apply artificial intelligence (AI) to achieve automatic diagnosis and visualization of early ONFH, thereby improving the success rate of hip-preserving treatments.

**Method:**

This retrospective study constructed a multicenter dataset using MRI data of 381 femoral heads from 209 patients with ONFH collected from four institutions (including 239 early ONFH cases and 142 non-ONFH cases). The dataset was divided into training, validation, and internal and external test datasets. This study developed a 3D ensemble framework to automatically diagnose early osteonecrosis of the femoral head based on MRI and utilized 3D Grad-CAM to visualize its decision-making process. Finally, the diagnostic performance of the framework was experimentally evaluated on the MRI dataset and compared with the diagnostic results of three orthopedic surgeons.

**Results:**

On the internal test dataset, the 3D-ONFHNet framework achieved overall diagnostic performance with an accuracy of 93.83%, sensitivity of 89.44%, specificity of 95.56%, F1-score of 87.67%, and AUC of 95.41%. On the two external test datasets, the framework achieved overall diagnostic accuracies of 87.76% and 87.60%, respectively. Compared to three orthopedic surgeons, the diagnostic performance of the 3D-ONFHNet framework was comparable to that of senior orthopedic surgeons and superior to that of junior orthopedic surgeons.

**Conclusions:**

The framework proposed in this study can generate staging results for early ONFH and provide visualizations of internal signal changes within the femoral head. It assists orthopedic surgeons in screening for early ONFH on MRI in a clinical setting, facilitating preoperative planning and subsequent treatment strategies. This framework not only enhances diagnostic efficiency but also offers valuable diagnostic references for physicians.

## 1 Introduction

Osteonecrosis of the femoral head (ONFH) is a refractory and disabling disease that commonly occurs in adults aged 20–40 years (1). The therapeutic outcome of ONFH depends on the stage of intervention (2). If not treated promptly in the early stages, it can lead to femoral head collapse, ultimately requiring treatments such as total hip arthroplasty (3, 4). However, after total hip arthroplasty, not only is hip function poor, but there are also complications like infection and prosthesis loosening (5, 6). Therefore, for young and middle-aged patients, to avoid the harm caused by total hip arthroplasty, hip-preserving surgery is the preferred option (7–9). Early diagnosis of the ONFH and precise staging of ONFH are important clinical goals that can improve the success rate of hip-preserving treatments.

As the treatment of ONFH is closely related to staging diagnosis, an ideal ONFH staging system can achieve reliable prognostic evaluations and effective treatment plans (10). The modified Ficat-Arlet staging system, as one of the most commonly used staging systems (11, 12), divides ONFH into stages 0 to IV. The Ficat-Arlet staging system provides important diagnostic evidence for selecting surgical intervention plans and can enhance the effectiveness of hip-preserving treatments before stage III. Clinical data show that compared to stage II, patients at stage III have significantly lower hip joint function scores after hip-preserving surgery. In this study, stages 0 to II are considered the early stages of ONFH (13, 14). Currently, MRI is regarded as the gold standard for detecting early ONFH (2, 15), featuring high sensitivity and specificity. It can address the issue of early ONFH not showing obvious manifestations on x-ray images, helping orthopedic surgeons promptly diagnose early ONFH. Therefore, precise and fine-grained staging of early ONFH enables orthopedic surgeons to promptly intervene within the treatment window, developing treatment plans such as minimally invasive surgeries based on the extent of osteonecrosis to prevent further progression of osteonecrosis. This is crucial for the treatment and prognosis of patients.

Artificial intelligence algorithms can assist in the diagnosis of diseases and to some extent reach the diagnostic level of human experts. For example, intelligent diagnosis of bone infections based on medical images such as MRI or CT (16, 17), and automatic detection of fractures (18, 19). However, to our knowledge, there is no 3D deep learning model that automatically diagnoses MRI images of early ONFH based on the modified Ficat-Arlet staging system. Moreover, the diagnostic task of early ONFH based on MRI faces the following challenges: (1) MRI three-dimensional volumetric data contains multi-planar features and other three-dimensional characteristics. Performing feature analysis only on MRI slices would lose the diagnostic basis for osteonecrosis contained in the three-dimensional features (such as spatial information and morphological information) (20–22). The data characteristics of MRI pose challenges for the model's ability to process three-dimensional volumetric data. (2) Compared to the characteristics of ONFH after collapse, the features of early ONFH (before collapse) are subtler, requiring deep networks to comprehensively analyze key pathological features such as abnormal signal regions within the femoral head and differences in the nature of edema. (3) Deep learning models for early ONFH lack research on multicenter datasets, and their generalizability and robustness in clinical settings need further evaluation.

Based on the above challenges, this study aims to develop and evaluate an MRI-based 3D ensemble artificial intelligence framework as an auxiliary diagnostic tool for the automatic diagnosis and fine-grained staging of early ONFH. This framework will support the creation of personalized treatment plans for diverse patients in clinical settings.

## 2 Material and methods

This retrospective study was approved by the Institutional Review Board [IRB 2022-KY-101(K)-(1)], and the requirement for informed consent was waived. All experiments were performed in accordance with relevant ethical guidelines and regulations. The study adhered to the principles of the Declaration of Helsinki. Additionally, all participating hospitals/institutions were informed and consented to the study protocol.

### 2.1 Patient selection

The MRI images used in this multicenter study were obtained from four different clinical centers between February 2018 and September 2022: (A) Shanghai Sixth People's Hospital Affiliated to Shanghai Jiaotong University School of Medicine, (B) Sanmenxia Central Hospital, (C) Jiashan First People's Hospital in Zhejiang Province, and (D) Xinghua Traditional Chinese Medicine Hospital. All multicenter data were anonymized for analysis.

The study dataset consisted of hip MRI data from patients aged 18 years and older, including axial, coronal, and sagittal T2-weighted images (T2WI). Inclusion criteria included patients with early ONFH assessed based on the Ficat-Arlet staging system (stages 0-II). Data from patients who had undergone total hip arthroplasty, poor image quality or low signal-to-noise ratio in hip MRI images, or congenital hip dysplasia were excluded. The early ONFH group included all MRI data diagnosed as stages I and II, while the non-ONFH group consisted of MRI data of normal femoral heads (stage 0). This inclusion criterion enhanced the model's ability to adapt to actual clinical settings.

### 2.2 MRI acquisition

All patients in the multicenter dataset underwent Magnetic Resonance Imaging (MRI) examinations. The MRI scans were performed using MAGNETOM Prisma 3T MRI systems (Siemens Healthcare, Erlangen, Germany) and Philips Ingenia 1.5T MRI systems (Philips, Best, The Netherlands) to obtain MRI volumes from four hospitals. During the MRI examinations, patients were positioned supine with both lower limbs kept symmetrical and toes adducted and internally rotated. The imaging range for bilateral hip joint MRI plain scans typically extended superiorly to the anterior superior iliac spine and inferiorly below the lesser trochanter of the femur. In the anterior-posterior view, the central beam was aligned with the midpoint between the upper edge of the pubic symphysis and the horizontal line connecting the anterior superior iliac spines on both sides, and the coronal scan covered the entire femoral head. In the axial, coronal, and sagittal planes, Spin-Echo (SE) T1-weighted and Fast SE T2-weighted sequences were used for imaging. All hip MRI data were extracted from hospitals in Digital Imaging and Communications in Medicine (DICOM) format, exported to personal computers and subsequently anonymized.

### 2.3 Dataset generation

The MRI dataset was annotated by two orthopedic surgeons with over 10 years of clinical experience using ITK-SNAP software (v3.8.0) to annotate bounding boxes of the core area of the femoral head and the staging results. The staging annotations were derived by integrating MRI and x-ray medical images. Due to the difficulty in annotating early ONFH, each expert independently completed the annotations and then compared the results. For images with annotation discrepancies, the final annotation was determined through consensus discussion and majority voting.

**Figure 1:**
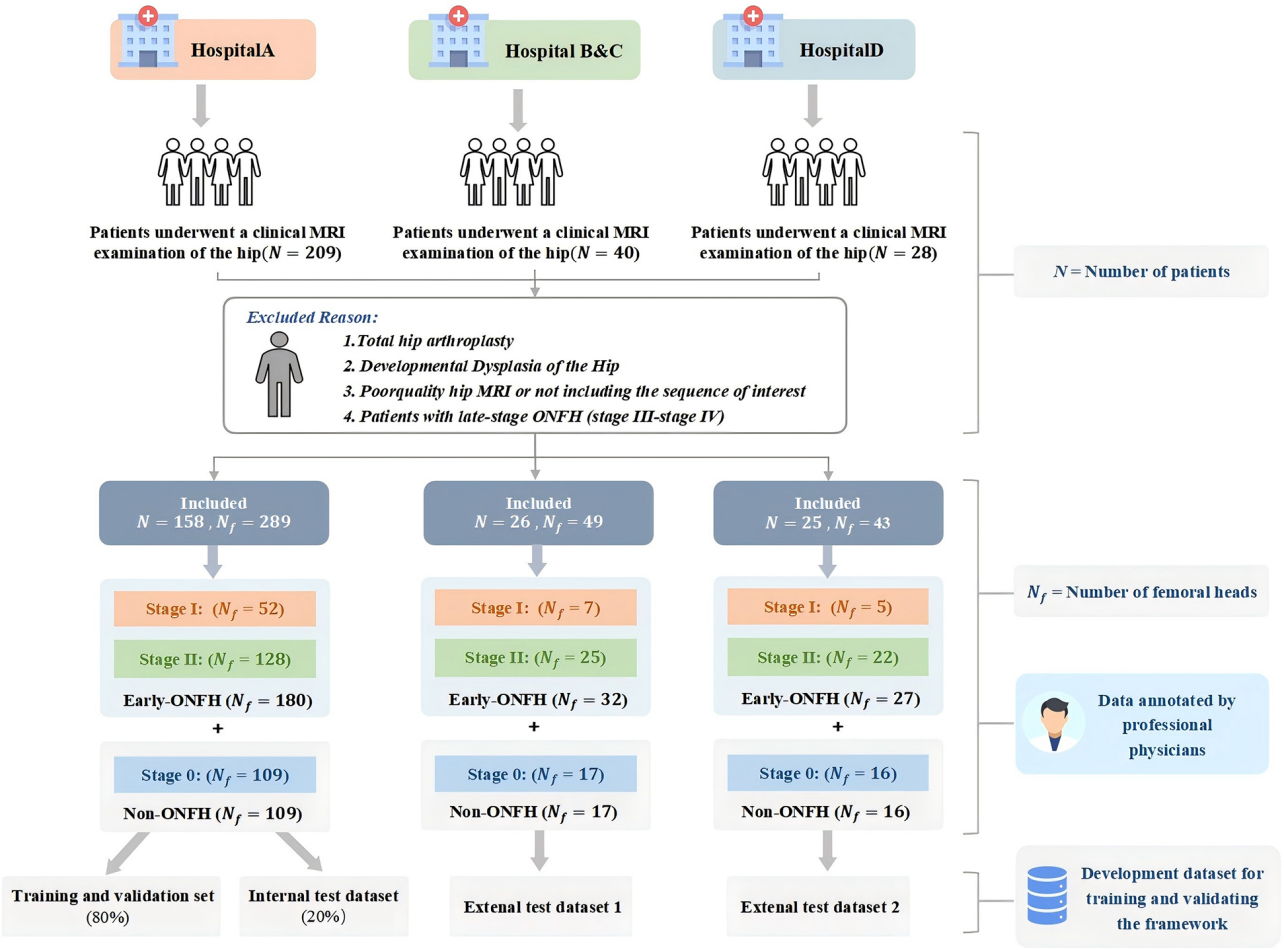
Multicenter data composition diagram.

The dataset construction process and detailed exclusion criteria are shown in Figure 1. Patients from Hospital A were used as the internal dataset, which was divided into training, validation, and internal test datasets for training and validating the model. Patients from Hospitals B, C, and D were divided into two external test datasets based on MRI scanners to evaluate model performance and assess the model's generalization ability.

### 2.4 Model development

**Figure 2:**
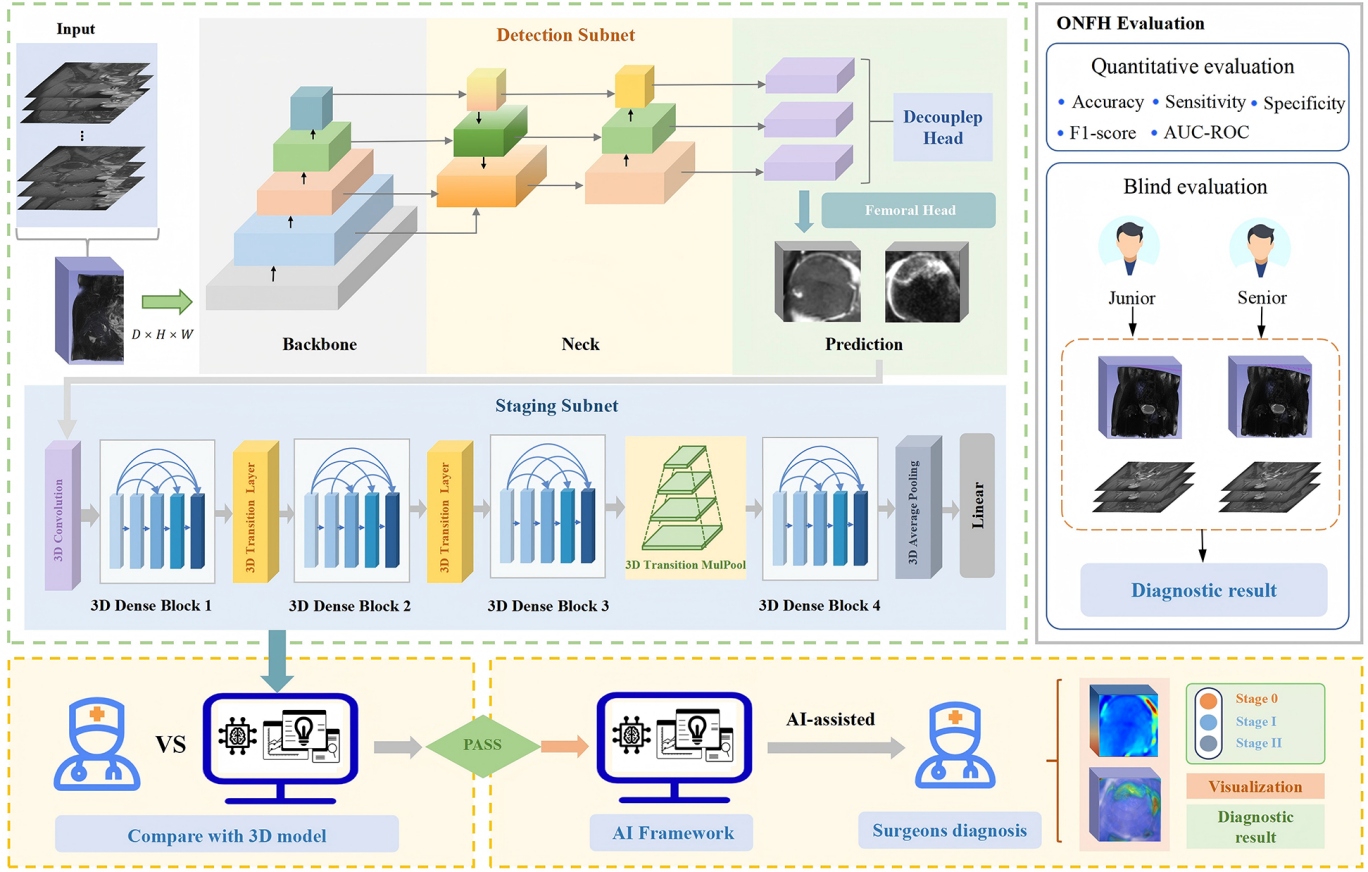
The overall 3D ensemble framework proposed in this study, 3D-ONFHNet. 3D, three-dimensional; ONFH, osteonecrosis of the femoral head.

Figure 2 shows the composition and prediction process of our proposed 3D ensemble framework, 3D-ONFHNet, which achieves accurate detection of the core area of the femoral head, diagnosis of early osteonecrosis of the femoral head (ONFH), fine-grained staging of early ONFH, and visualization tasks. In the first stage, the three-dimensional detection subnet 3DC-YOLOX locates the core area of the femoral head from the three-dimensional MRI volumetric data of the hip. This enables the staging subnet in the second stage to focus on the femoral head region for feature extraction, reducing interference from irrelevant regions during feature analysis. In the second stage, based on the femoral head core area localized by the detection subnet, the staging subnet 3D-MulDenseNet diagnoses whether the input femoral head has early ONFH and obtains fine-grained staging results for early ONFH.

**Figure 3:**
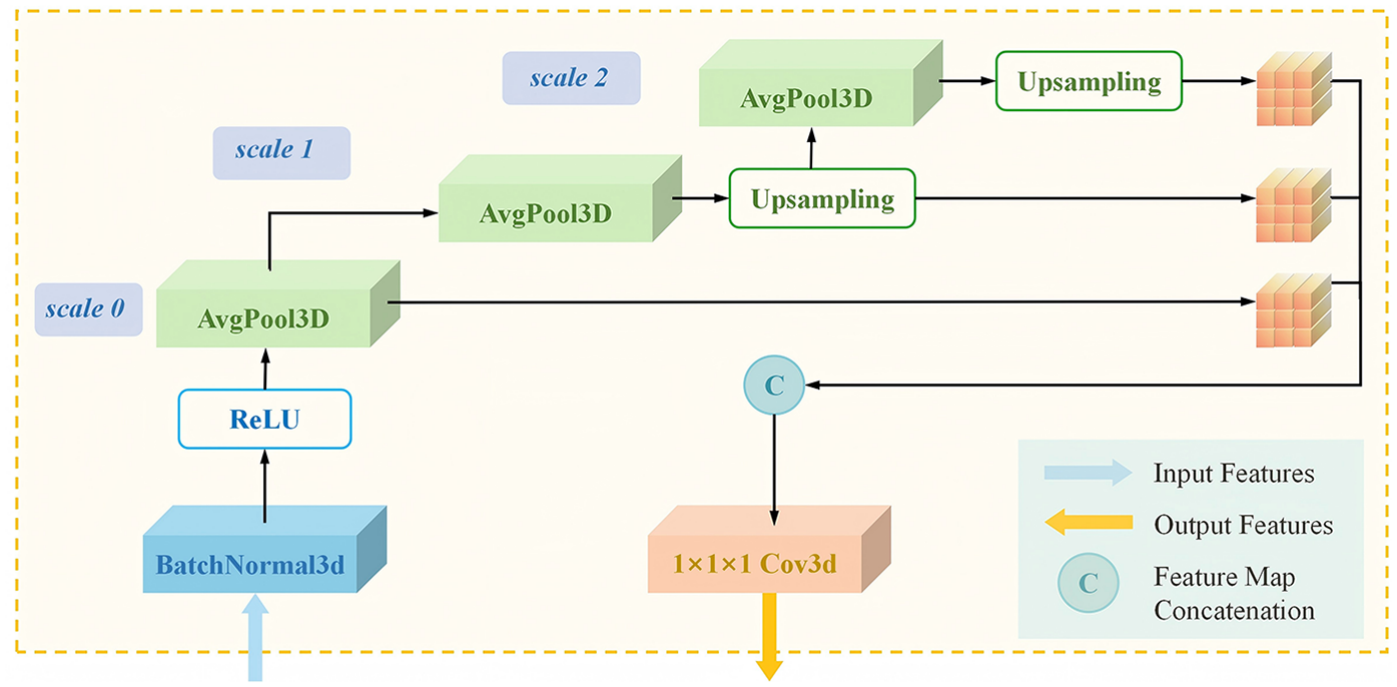
Architecture of the 3D transition multiPool module in the staging subnet. 3D, three-dimensional.

To enhance the ability of the staging subnet to to extract and analyze MRI features, we proposed the MulPoolFusion module (Figure 3) for multi-scale analysis, thereby extracting both local and global features of the femoral head and further improving the accuracy of early ONFH staging. Therefore, compared with classical 2D CNN models, the staging subnet 3D-MulDenseNet can fully utilize the continuous spatial information and morphological information of three-dimensional volumetric data to automatically diagnose lesion conditions within the entire femoral head.

An improved 3D Grad-CAM method is applied to the last three-dimensional convolutional layer of the staging subnet to generate internal visualization results of the femoral head for the test dataset. The visualization results provide information on signal changes within the femoral head and can be used to create three-dimensional reconstructed views that assist orthopedic surgeons in diagnosing the progression of ONFH and conducting preliminary screenings in clinical settings.

### 2.5 Model training

**Figure 4:**
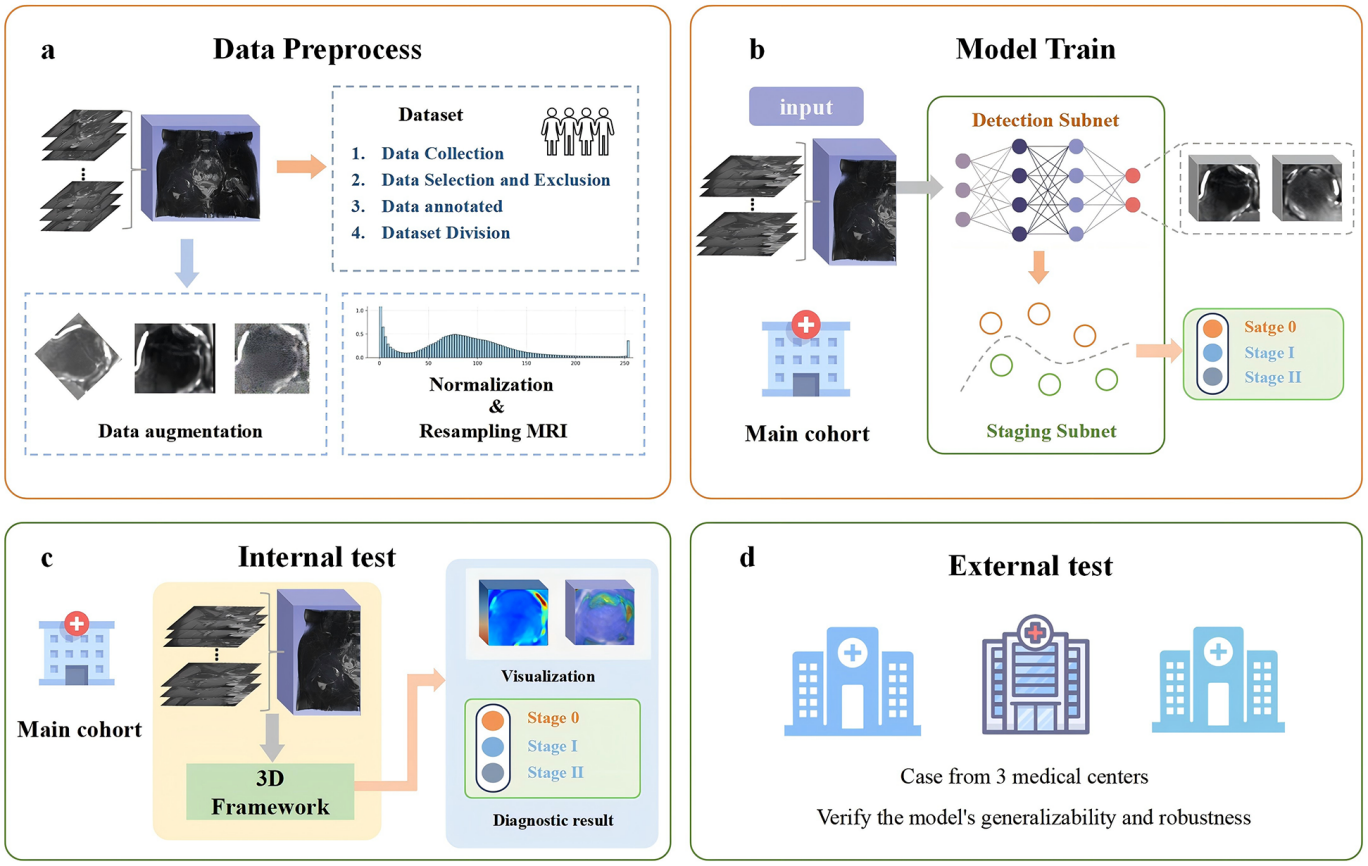
Overview of the experimental workflow. **(a)** Data preprocessing: the collected raw data are preprocessed to ensure consistent data quality. **(b)** Model training: the preprocessed data are then provided as input to the deep learning model, and the model parameters are optimized to achieve the best possible performance. **(c)** Internal testing: the trained model is evaluated using internal test datasets to assess its performance. **(d)** External testing: external datasets are employed to validate the model's generalizability and robustness.

Figure 4 presents the complete process of framework development. To ensure better detection performance and reduce the risk of overfitting when processing three-dimensional volumetric MRI data of the hip, we performed data preprocessing on the MRI images before training. First, all hip MRI images were resampled and centrally cropped. Then, the cropped MRI images were resized uniformly to 32 × 112 × 64 pixels, and their pixel values were normalized with a window level of 300 and a window width of 1,500. Additionally, to address data imbalance and enhance the model's ability to handle noise and bias present in real clinical settings, data augmentation techniques such as random brightness, random flipping, and Gaussian noise were applied to the training set. These methods improve the model's generalization and help prevent overfitting.

The 3D-ONFHNet framework was developed using PyTorch (version 1.7.0, https://pytorch.org) on a 32GB NVIDIA Tesla V100. In the staging subnet, Focal Loss was used as the loss function, and the Adam optimizer with weight decay was used for training with a batch size of 100 and a learning rate of 1e-2. The framework adopts a two-stage training strategy. First, after obtaining the optimal femoral head localization model in the first stage of training, the femoral head data localized in this stage was used to generate the training and validation sets for the early ONFH diagnosis task in the second stage. Finally, the optimal predictive models trained in the two subnets were integrated into an ensemble framework.

### 2.6 Comparison of diagnostic performance with orthopedic surgeons

For the internal and external test datasets, evaluations were conducted by two junior orthopedic surgeons with three and five years of clinical experience, respectively, and one senior orthopedic surgeon with over ten years of clinical experience. They classified each femoral head as early ONFH or non-ONFH and additionally subclassified early ONFH into stage I or stage II. The test datasets provided to the surgeons contained only images with other information masked, ensuring that the three surgeons independently performed diagnosis and evaluation.

### 2.7 Statistical analysis

Statistical analysis was performed using SPSS software (version 21.0), with statistical significance set at *p* < 0.05. The McNemar test was used to compare performance differences between the predictions of the 3D-ONFHNet framework and the diagnoses made by orthopedic surgeons. To evaluate the fine-grained staging results of early ONFH, the accuracy, sensitivity, specificity, F1 score, area under the curve (AUC) were used. Macro-average and weighted average metrics were used to assess overall diagnostic performance. The macro-average is calculated by averaging the metrics computed independently for each category, while weighted average is calculated by giving different weights to each category based on the number of samples in that category, providing a more accurate reflection of the model's performance in practical applications.

## 3 Result

### 3.1 Characteristics of included participants

The dataset of this study comprised 381 MRI scans of the femoral head from 209 participants recruited between February 2018 and September 2022 from four hospitals. Among these, 142 cases were diagnosed with early ONFH, and 239 cases were non-ONFH. The average age of participants was 52.5 years (ranging from 18 to 68 years), with 56.17% male and 43.83% female. Participants from Shanghai Sixth People's Hospital, affiliated with Shanghai Jiao Tong University School of Medicine, were designated as the internal dataset. The internal dataset was split into training, validation and an internal test dataset at an 8:1:1 ratio. The datasets from the other three clinical centers were divided into two external independent test datasets based on the MRI scanners used, which were utilized to evaluate model performance and assess the framework's generalization ability (Figure 1).

### 3.2 Diagnostic performance of 3D-ONFHNet

The performance of the trained and optimized 3D-ONFHNet framework for early ONFH staging was evaluated using both internal and external test datasets. In the internal test dataset, the 3D-ONFHNet framework achieved macro-average values for overall diagnostic accuracy, sensitivity, specificity, F1-score, and AUC of 93.83%, 89.44%, 95.56%, 87.67%, and 95.14%, respectively. Additionally, the framework demonstrated high accuracy in the fine-grained staging of early ONFH, with stages 0 and II showing the highest AUCs (98.19% and 96.81%) (Table 1). The macro-average and weighted average AUCs of the 3D-ONFHNet framework reached 95%, indicating that the proposed framework performs excellently in comprehensive diagnostic tasks for early ONFH.

**Table 1:** The dignostic performance of 3D-ONFHNet framework for early osteonecrosis of the femoral head in the internal test dataset.

Parameter	Ficat-Arlet system			Macroaverage	Weighted average
	Stage 0	Stage I	Stage II		
Accuracy (%)	94.44	92.59	94.44	93.83	94.17
Sensitivity (%)	87.50	87.50	93.33	89.44	90.74
Specificity (%)	97.37	93.47	95.83	95.56	95.94
F1-score (%)	90.32	77.77	94.92	87.67	91.02
AUC (%)	98.19	87.77	96.81	95.14	95.87

**Figure 5:**
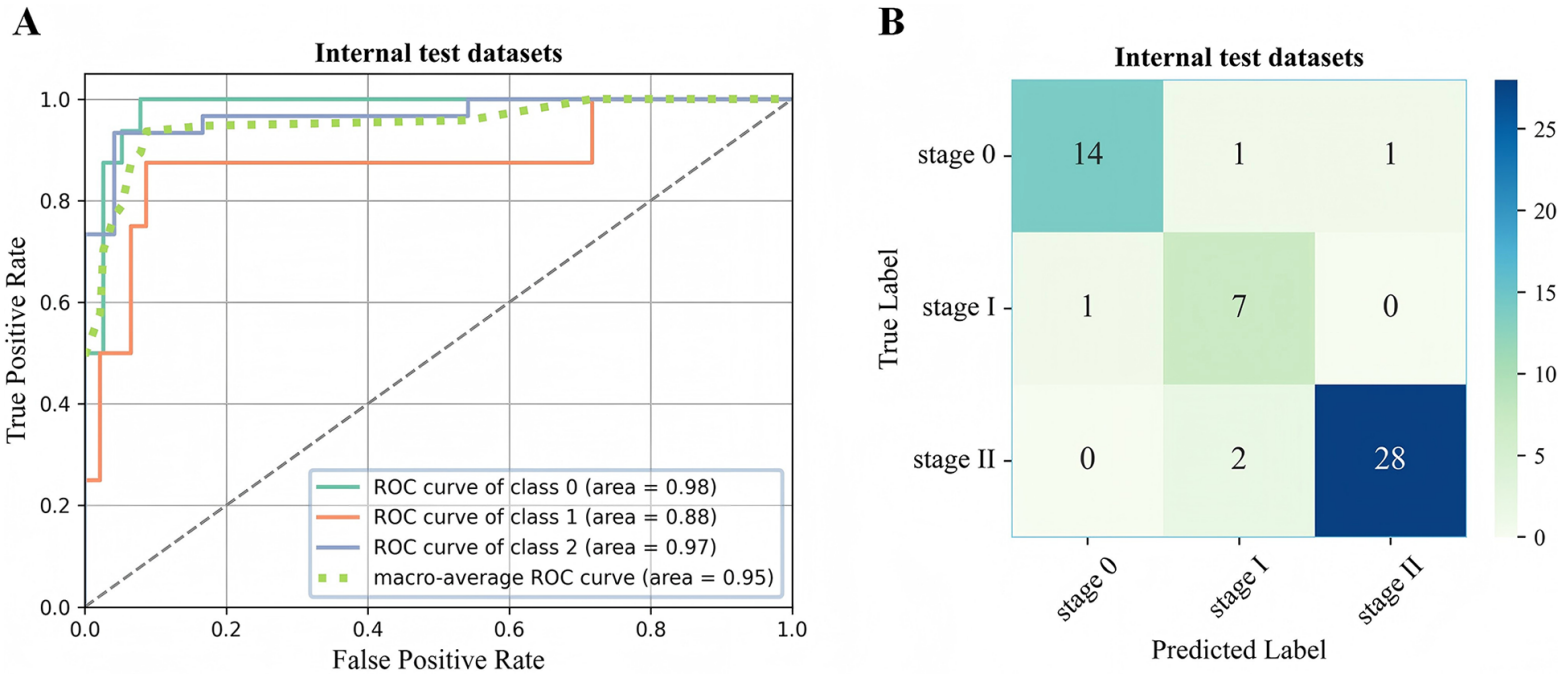
ROC curve **(A)** and confusion matrix **(B)** for evaluating the staging performance of the 3D-ONFHNet framework in the internal test dataset. ROC, receiver operating characteristic.

**Figure 6:**
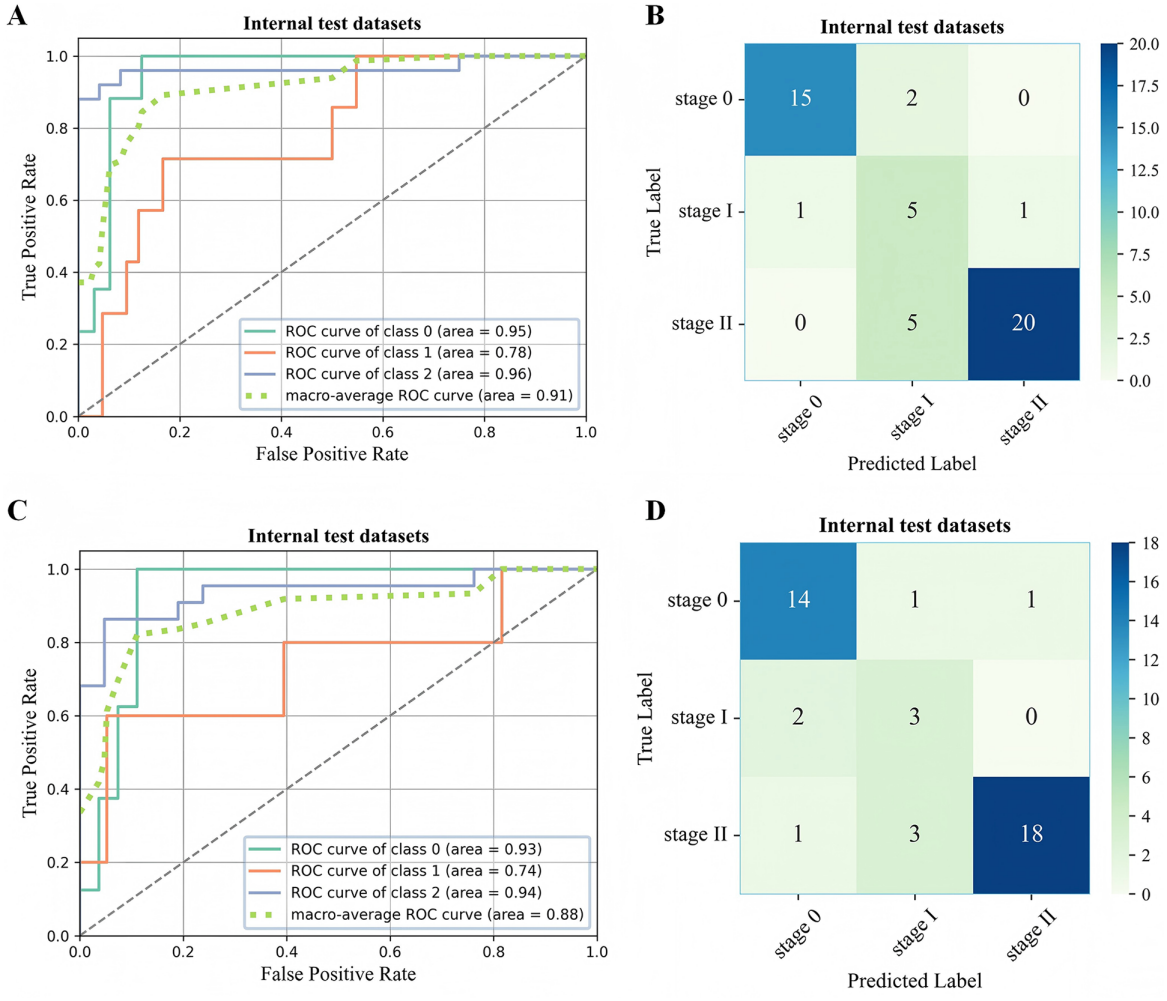
ROC curve **(A,C)** and confusion matrix **(B,D)** for evaluating the staging performance of the 3D-ONFHNet framework in the external test dataset. ROC, receiver operating characteristic.

In the two external test datasets, the 3D-ONFHNet framework achieved macro-average values for overall accuracy, sensitivity, specificity, and F1-score of 87.76%, 79.89%, 92.02%, 76.83% and 89%, 81.63%, 94.44%, 83.42%, respectively (Table 2). Figures 5 and 6 present the receiver operating characteristic (ROC) curves and confusion matrices for different stages of ONFH (stages 0-II) in the internal and external datasets, visually demonstrating the excellent performance of our model in the diagnostic tasks for early ONFH.

**Table 2:** The dignostic performance of 3D-ONFHNet for early osteonecrosis of the femoral head in the external test datasets.

Dataset	Parameter	Ficat-Arlet system			Macroaverage	Weighted average
		Stage 0	Stage I	Stage II		
External test dataset 1	Accuracy (%)	93.87	81.63	87.76	87.76	89.00
	Sensitivity (%)	88.24	71.43	80.00	79.89	81.63
	Specificity (%)	96.87	83.33	95.83	92.01	94.44
	F1-score (%)	90.91	52.63	86.96	76.83	83.42
	AUC (%)	94.85	78.23	96.49	91.29	93.31
External test datase 2	Accuracy (%)	88.37	86.04	88.37	87.60	88.10
	Sensitivity (%)	87.50	60.00	81.82	76.44	81.39
	Specificity (%)	88.88	89.47	95.24	91.20	92.21
	F1-score (%)	84.85	50.00	87.80	74.33	82.31
	AUC (%)	93.06	73.68	93.72	88.43	91.14

### 3.3 Comparison of performance of the ensemble framework and orthopedic surgeons

The 3D-ONFHNet framework was compared with three orthopedic surgeons based on internal and external test datasets to evaluate the framework's effectiveness and applicability in real clinical settings. Table 3 shows the performance differences between the framework's predictions and the orthopedic surgeons' diagnoses. The McNemar test results indicate that the 3D-ONFHNet framework performs comparably to senior orthopedic surgeons in diagnosing early ONFH but is outperforms junior orthopedic surgeons (Table 3). In the internal dataset, the overall diagnostic performance of the 3D-ONFHNet framework improved compared to the three orthopedic surgeons and significantly outperformed the junior orthopedic surgeons in the external test datasets.

**Table 3:** The performance comparison of orthopedic surgeons and the 3D-ONFHNet framework for the diagnosis of early osteonecrosis of the femoral head on the internal and the external validation datasets.

Dataset	DL/surgeons	Macroaverage accuracy	Macroaverage sensitivity	Macroaverage specificity	Macroaverage F1-score	*P*-value
Internal test dataset 1	DL model	93.83	89.44	95.56	87.67	–
	Senior	89.51	77.92	92.06	78.50	.218
	Junior					
	#1	81.48	68.19	87.92	66.69	<.001
	#2	82.72	69.31	88.13	68.49	.002
External test dataset 1	DL model	87.76	79.89	92.01	76.83	–
	Senior	88.44	75.13	93.35	76.18	.360
	Junior					
	#1	78.23	61.82	84.19	61.06	0.007
	#2	80.95	63.15	87.40	63.87	0.015
External test dataset 2	DL model	87.60	76.44	91.20	74.33	–
	Senior	86.05	72.27	91.91	71.14	.250
	Junior					
	#1	78.29	64.70	85.75	61.76	<.001
	#2	81.40	65.64	89.45	65.16	.002

### 3.4 Model visualization via 3D Grad-CAM

**Figure 7:**
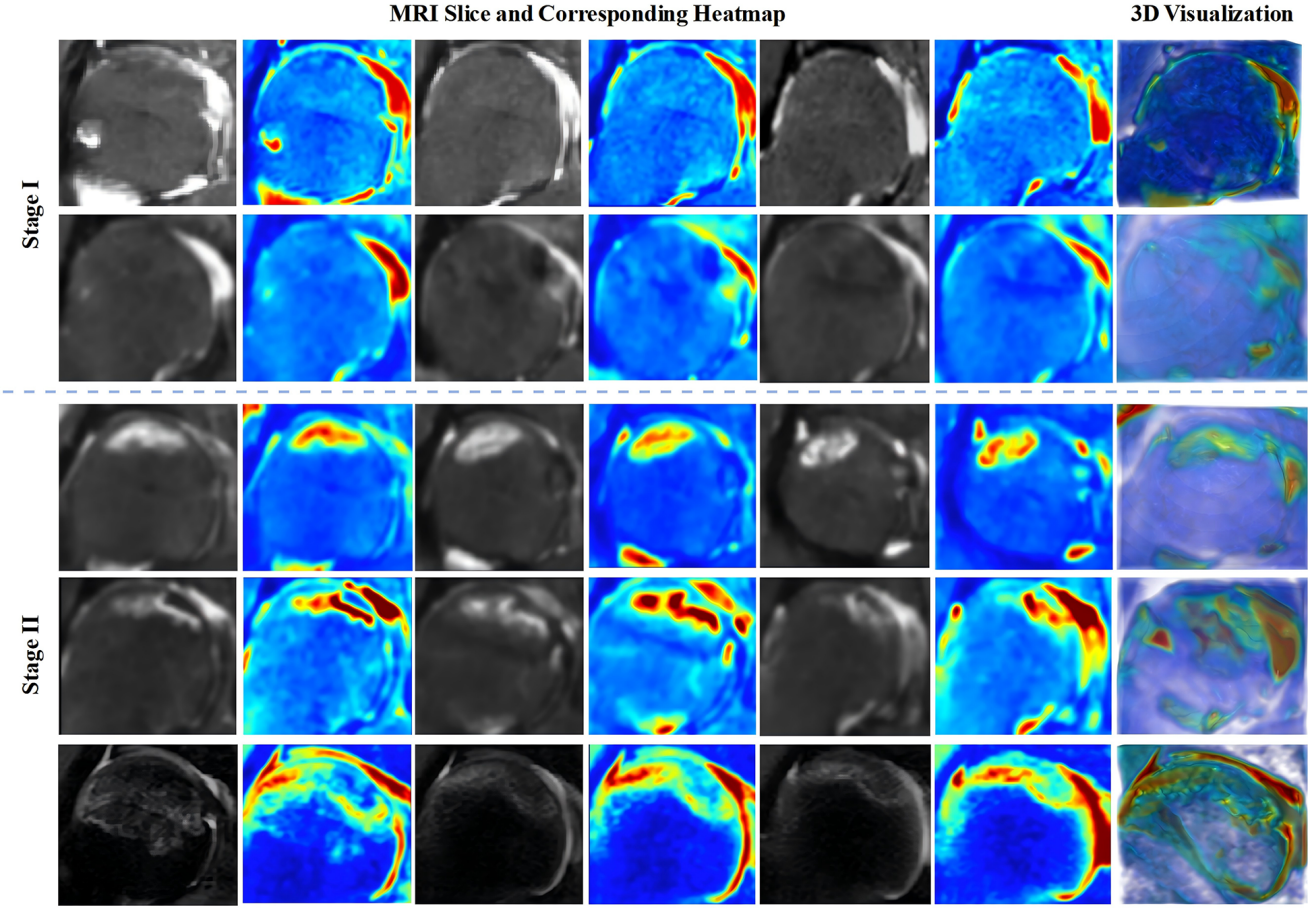
Visualization of 3D heatmaps for stage I-II ONFH and 3D reconstructed heatmap images. Key slices of the femoral head are selected for display in the 3D heatmap visualization. 3D, three-dimensional; ONFH, osteonecrosis of the femoral head.

To enhance the interpretability of the framework's decisions and better address the needs of real clinical settings, we employed the 3D Grad-CAM method to generate visualizations of the MRI data for the femoral head. These visualizations are used to observe abnormal regions within the femoral head, where signal changes occur. Additionally, by reconstructing these visualizations of the femoral head using three-dimensional reconstruction tools, the volume and other morphological characteristics of the abnormal regions can be further assessed (Figure 7). Therefore, these visualizations not only improve the interpretability of the framework but also provide precise visual aids for orthopedic surgeons when diagnosing early ONFH, thereby enhancing the efficiency and accuracy of clinical diagnoses.

## 4 Discussion

In this multicenter study, we proposed a 3D ensemble framework for the auxiliary diagnosis of early osteonecrosis of the femoral head (ONFH), called 3D-ONFHNet. Aligned with the diagnostic process of orthopedic surgeons, the framework automatically detects the core region of the femoral head from hip MRI data, provides diagnostic results for early ONFH and visualizing internal signal changes within the femoral head. Experimental results from both internal and external test datasets demonstrate that 3D-ONFHNet achieves diagnostic performance comparable to senior orthopedic surgeons and significantly outperforms junior orthopedic surgeons. These findings validates the feasibility and generalizability of 3D-ONFHNet in clinical settings. 3D-ONFHNet provides strong support for the early diagnosis of ONFH and facilitates hip-preserving surgery.

Many neural network models have been developed and applied to research in fields such as quantitative assessment and assisted diagnosis based on medical images (23–25). However, research on using deep learning techniques for staging early ONFH to assist in surgical planning remains relatively limited. Kim et al. (26) used anteroposterior pelvic x-rays as input data and, based on EfficientNet and Xception models, achieved diagnosis of ONFH before collapse with an AUC of 0.902–0.912. Shen et al. (27) conducted a single-center retrospective study using 11,061 MRI slices to train and optimize a CNN model, achieving an AUC of 0.98 in identifying early ONFH (before the IIIA stage of the Association Research Circulation Osseous staging system). Li et al. (28) used the SRANet model to perform a classification task on 1,367 CT images distinguish between AVNFH absence and AVNFH presence classes, achieving a classification performance with an AUC of 0.95. Most models are based on single-center datasets to achieve binary classification tasks between ONFH and Non-ONFH or between early-stage and late-stage ONFH. However, in clinical settings, compared to diagnosing whether a patient has ONFH, fine-grained early ONFH staging can help orthopedic surgeons detect lesions early and intervene promptly within the treatment window. This enables the development of conservative treatment plans or the scheduling of hip-preserving procedures based on the fine-grained staging results, ultimately preventing further expansion of the irreversible osteonecrosis area in the femoral head. Therefore, it holds greater practical value for disease prevention and clinical application (29, 30). Additionally, unlike other models that require manual segmentation of the femoral head region from the hip for result prediction, our framework can directly detect the core region of the femoral head and provide accurate diagnoses from hip MRI data.

Our framework aims to provide a more comprehensive assessment of early ONFH. It not only enables the diagnosis of early ONFH and Non-ONFH but also finely stages early ONFH cases into stages I and II, assisting orthopedic surgeons in intervening early to improve the success rate of hip-preserving treatments. It can be seen that current research on early ONFH diagnosis primarily relies on 2D MRI slice models, neglecting the spatial dimensional information contained in MRI as three-dimensional volumetric data. In contrast, our model uses complete three-dimensional volumetric data as input, preserving spatial features while providing richer feature representations, aligning with the clinical diagnostic methods of orthopedic surgeons. Additionally, to assist orthopedic surgeons in screening for early ONFH and evaluating the osteonecrosis area to prevent its progression and formulate treatment plans in a timely manner, as shown in Figure 7, we accurately depict the internal signal changes of the femoral head based on three-dimensional volumetric data using an improved 3D Grad-CAM method. Furthermore, after performing three-dimensional reconstruction of the heatmaps generated by this method, osteonecrosis area can be observed more intuitively. On the other hand, these studies based only on single-center datasets may lead to models lacking the ability to handle real clinical settings (31). In our study, we conducted rigorous external validation and detailed analysis using the multicenter dataset to evaluate the generalization and robustness of the proposed 3D-ONFHNet framework.

In summary, this study not only validates the feasibility and robustness of 3D-ONFHNet across multicenter datasets, but also introduces a novel approach for the precise diagnosis of early ONFH. By conducting fine-grained staging of early ONFH and providing visualizations of internal signal changes within the femoral head, it assists orthopedic surgeons in promptly identifying early ONFH and seizing the optimal window for hip-preserving treatments. Compared with traditional manual image review, this framework significantly reduces the workload of orthopedic surgeons in MRI screening and diagnosis, lowers the likelihood of missed diagnoses, and supports the development of subsequent personalized treatment plans. In clinical practice, this framework enables orthopedic surgeons to more efficiently screen large numbers of early ONFH cases, saving both clinical time and human resources. By facilitating earlier intervention, it also helps improve the overall success rate of hip-preserving surgeries, thereby reducing the future need for hip replacements and lowering the risk of related complications. Additionally, 3D-ONFHNet can accumulate sample data from early cases during diagnosis, which can be used for more precise prognostic assessments in subsequent medical research. These data support further clinical trials and the assessment of novel treatments for early ONFH, including new medications and surgical techniques.

However, our study has several limitations. First, although the research is based on a multicenter dataset, the sample size is relatively small, particularly for stage I data. Additionally, dataset includes special cases such as bone marrow edema and metallic implants. These unique samples are important for the application of the framework in real clinical settings, but future studies should increase the number of these samples to enhance the framework's generalization ability in handling complex clinical data. Second, diagnostic methods for ONFH also include x-rays and CT scans, which are equally essential imaging techniques for the accurate diagnosis of ONFH. Future research should explore multimodal approaches to comprehensively improve the diagnostic performance for ONFH. Third, since our framework employs a 3D deep learning model to process three-dimensional volumetric data, the training process requires a substantial amount of memory and computational resources. Our long-term goal is to deploy 3D-ONFHNet as an AI-assisted tool in real clinical settings. Therefore, future work should focus on further optimizing the framework to facilitate easier deployment and reduce resource consumption, thereby providing orthopedic surgeons with real-time, convenient diagnostic support. For the future development direction of this study, through the continuous accumulation of patient data and architectural upgrading, we aim to establish a fully automated AI solution capable of generating both diagnostic reports and treatment plans for osteonecrosis of the femoral head. This integrated approach will make a substantial contribution to the standardized diagnosis and treatment of early ONFH, as well as to research on long-term clinical outcomes.

## 5 Conclusion

In conclusion, this study proposes the 3D ensemble framework, 3D-ONFHNet, which is trained and validated on a multicenter MRI dataset. It effectively assists in the diagnosis and fine-grained staging of early osteonecrosis of the femoral head, while offering clinical interpretability. This fully automated diagnostic framework can significantly aids orthopedic surgeons in screening for early osteonecrosis of the femoral head, preventing the oversight of potential lesions and enabling timely intervention that improve the success rate of hip-preserving surgery.

## Data Availability

The raw data supporting the conclusions of this article will be made available by the authors, without undue reservation.
